# Chemical Nature of Heterogeneous Electrofreezing of Supercooled Water Revealed on Polar (Pyroelectric) Surfaces

**DOI:** 10.1021/acs.accounts.2c00004

**Published:** 2022-05-03

**Authors:** Leah Fuhrman Javitt, Sofia Curland, Isabelle Weissbuch, David Ehre, Meir Lahav, Igor Lubomirsky

**Affiliations:** Department of Molecular Chemistry and Materials Science, Weizmann Institute of Science, Rehovot 7610001, Israel

## Abstract

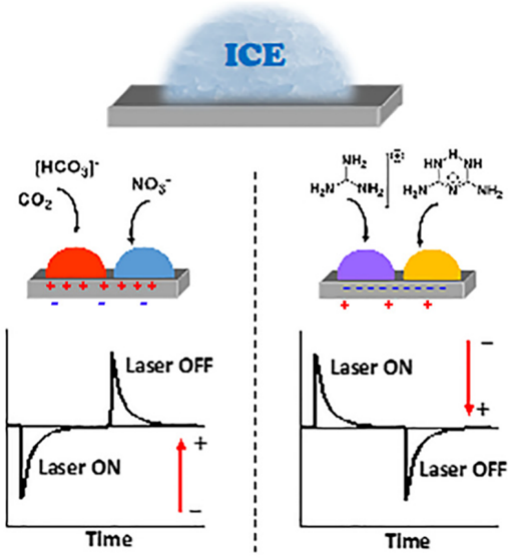

The ability to control the icing temperature of supercooled water (SCW) is of supreme importance in subfields of pure and applied sciences. The ice freezing of SCW can be influenced heterogeneously by electric effects, a process known as electrofreezing. This effect was first discovered during the 19th century; however, its mechanism is still under debate. In this Account we demonstrate, by capitalizing on the properties of polar crystals, that heterogeneous electrofreezing of SCW is a chemical process influenced by an electric field and specific ions. Polar crystals possess a net dipole moment. In addition, they are pyroelectric, displaying short-lived surface charges at their hemihedral faces at the two poles of the crystals as a result of temperature changes. Accordingly, during cooling or heating, an electric field is created, which is negated by the attraction of compensating charges from the environment. This process had an impact in the following experiments. The icing temperatures of SCW within crevices of polar crystals are higher in comparison to icing temperatures within crevices of nonpolar analogs. The role played by the electric effect was extricated from other effects by the performance of icing experiments on the surfaces of pyroelectric quasi-amorphous SrTiO_3_. During those studies it was found that on positively charged surfaces the icing temperature of SCW is elevated, whereas on negatively charged surfaces it is reduced. Following investigations discovered that the icing temperature of SCW is impacted by an ionic current created within a hydrated layer on top of hydrophilic faces residing parallel to the polar axes of the crystals. In the absence of such current on analogous hydrophobic surfaces, the pyroelectric effect does not influence the icing temperature of SCW. Those results implied that electrofreezing of SCW is a process influenced by specific compensating ions attracted by the pyroelectric field from the aqueous solution. When freezing experiments are performed in an open atmosphere, bicarbonate and hydronium ions, created by the dissolution of atmospheric CO_2_ in water, influence the icing temperature. The bicarbonate ions, when attracted by positively charged pyroelectric surfaces, elevate the icing temperature, whereas their counterparts, hydronium ions, when attracted by the negatively charged surfaces reduce the icing temperature. Molecular dynamic simulations suggested that bicarbonate ions, concentrated within the near positively charged interfacial layer, self-assemble with water molecules to create stabilized slightly distorted “ice-like” hexagonal assemblies which mimic the hexagons of the crystals of ice. This occurs by replacing, within those ice-like hexagons, two hydrogen bonds of water by C–O bonds of the HCO_3_^–^ ion. On the basis of these simulations, it was predicted and experimentally confirmed that other trigonal planar ions such as NO_3_^–^, guanidinium^+^, and the quasi-hexagonal biguanidinium^+^ ion elevate the icing temperature. These ions were coined as “ice makers”. Other ions including hydronium, Cl^–^, and SO_4_^–2^ interfere with the formation of ice-like assemblies and operate as “ice breakers”. The higher icing temperatures induced within the crevices of the hydrophobic polar crystals in comparison to the nonpolar analogs can be attributed to the proton ordering of the water molecules. In contrast, the icing temperatures on related hydrophilic surfaces are influenced both by compensating charges and by proton ordering.

## Key references

- Gavish, M.; Wang, J.; Eisenstein, M.; Lahav, M.; Leiserowitz, L. The role of crystal polarity in alpha-amino acid crystals for induced nucleation of ice.^1^*Science***1992**, *256*, 815–818. The anomaly of the induced ice nucleation by amino acids was resolved by demonstrating that crystal polarity influences ice nucleation.
- Ehre, D.; Lavert, E.; Lahav, M.; Lubomirsky, I. Water Freezes Differently on Positively and Negatively Charged Surfaces of Pyroelectric Materials.^2^*Science***2010**, *327*, 672–675. An unexpected positive negative effect in electrofreezing of supercooled water was discovered.
- Curland, S.; Javitt, L.; Weissbuch, I.; Ehre, D.; Lahav, M.; Lubomirsky, I. Heterogeneous Electrofreezing Triggered by CO_2_ on Pyroelectric Crystals: Qualitatively Different Icing on Hydrophilic and Hydrophobic Surfaces.^3^*Angew. Chem. Int. Ed.***2020**, *59*, 15570–15574. Atmospheric CO_2_ influences ice nucleation of supercooled water and explains the positive–negative effect.
- Curland, S.; Allolio, C.; Javitt, L.; Dishon, S.; Weissbuch, I.; Ehre, D.; Harries, D.; Lahav, M.; Lubomirsky, I. Heterogeneous Electrofreezing of Super-Cooled Water on Surfaces of Pyroelectric Crystals is Triggered by Planar-Trigonal Ions.^4^*Angew. Chem. Int. Ed.***2020**, *59*, 15574–15580. Electrofreezing is a cooperative process, which involves the interaction of an electric field with distinct ions.

## Introduction

No problem can be solved with the same consciousness that created it

Albert Einstein

Ice melts at 0 °C under atmospheric conditions; however, during the 18th century Fahrenheit discovered that water could be supercooled below 0 °C. When cooled under homogeneous conditions, liquid water may be cooled without freezing to about ∼−48 °C.^5^ The ability to control the freezing temperature of SCW is of topical importance, for example, for the glaciation of warm clouds for rain precipitation,^6^ the survival of cold-blooded animals in winter, the preservation of tissues for transplantation in medicine,^7^ the preservation of perishable food,^8^ etc.

The icing temperature of SCW can be controlled heterogeneously by electric fields.^9^ However, the mechanism of electrofreezing is still disputed.^10−17^ Several mechanisms suggested that formation of the ice-like nuclei is created by the alignment of water molecules along electric field lines,^18^ others suggest nucleation of water on top of gas bubbles,^19^ whereas others claim that icing is influenced by electric charges,^20−24^ etc.

Here, we describe icing experiments of SCW performed on surfaces of polar, pyroelectric crystals. These investigations resulted in the two following findings: a chemically driven icing process, which is influenced by the pyroelectric field and by compensating “ice-maker” or “ice-breaker” ionic charges. In addition, a proton-ordering process takes place within crevices of polar crystals that elevates the icing temperature of SCW.^2−4,14,21,25^

### Influence of Crystal Polarity on the Icing Temperature of Supercooled Water

**Figure 1:**
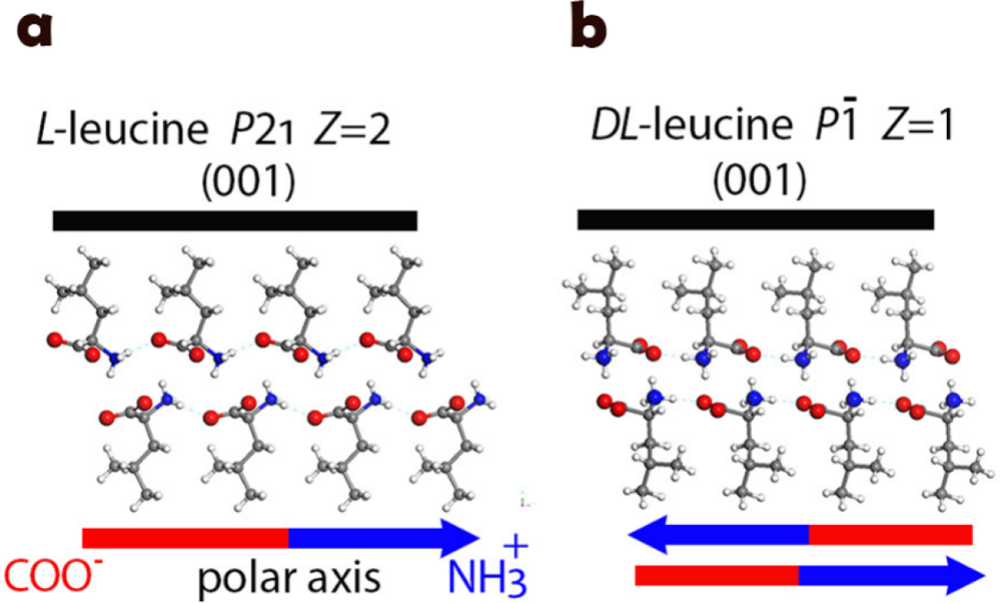
Packing arrangements of the investigated hydrophobic crystals expressing similar hydrophobic faces: (a) l-leucine crystals (space group *P*2_1_) and (b) dl-leucine crystals (space group *P*-1).

In 1947, Vonnegut demonstrated that crystals of AgI wurtzite polymorph can serve as efficient seeds for the glaciation of warm clouds for rain precipitation.^26^ As a result, an intense search for new materials, more environmentally friendly, followed. These investigations led to the understanding that other factors, apart from epitaxy, might elevate the icing temperature of SCW. Among these reports there was an unanticipated observation that powders of l-crystals of the hydrophobic α-amino acids can nucleate ice at temperatures that are higher by 3–5 °C in comparison to temperatures induced by powders of analogous racemic dl-crystals.^27,28^ None of these crystals displayed epitaxy with the ice crystals. Those results looked inexplicable since the chiral and the racemic hydrophobic α-amino-acid crystals express chemically identical crystalline faces, Figure 1.

**Figure 2:**
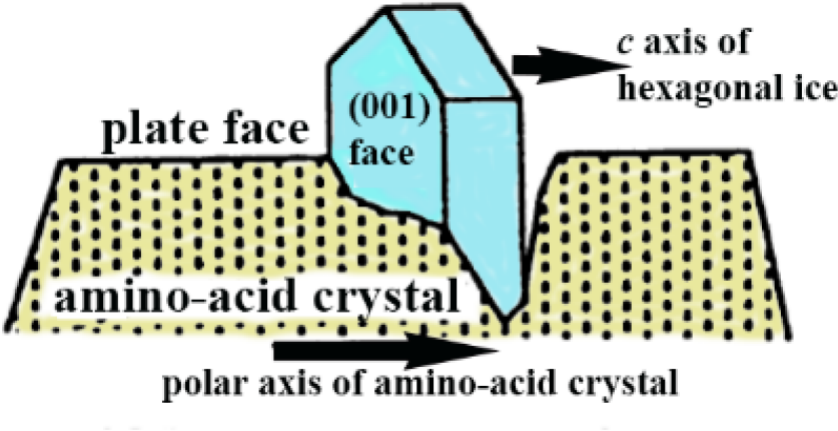
Ice crystal growing within the α-amino-acid crystal crack delineated by hydrophilic walls. Adapted with permission from ref (1). Copyright 2021 AAAS.

By performing icing experiments on the expressed faces of α-amino-acid single crystals, we found that ice nucleates and grows vertically within the hydrophilic cracks present on these faces (Figure 2).^1^

The surrounding surfaces that delineate these fissures are different in the two classes of crystals. The chiral crystals appear in polar space groups, whereas the racemates appear in centrosymmetric ones (Table 1). As a result of this difference in packing arrangement, the walls that delineate the crevices in the racemic crystals are identical, being composed from alternating layers of COO^–^ and NH_3_^+^ groups. In contrast, in the polar crystals, one wall exposes exclusively COO^–^ groups whereas the other one exclusively NH_3_^+^. These structural differences imply that the difference in icing temperature between the two classes of crystals is due to crystal polarity and not due to crystal chirality. Further confirmation of this deduction was provided by comparative icing experiments performed in parallel on surfaces of single crystals of hydrophilic dl-alanine and dl-tyrosine^1^ and in the different icing results of the two polymorphs of the l-isoleucine crystals.^3,30^ In contrast to the hydrophobic amino acids, the dl-crystals of alanine and tyrosine crystallize in the polar orthorhombic *Pna*2_1_ space group whereas the l- and d-enantiomers crystallize in the nonpolar, orthorhombic *P*2_1_2_1_2_1_ space group. The icing temperatures on the surfaces of the polar racemic crystals were ∼4–5 °C higher than those measured on the surfaces of the nonpolar enantiomorphs (Table 1).

**Table 1:** Freezing Points (FP) of Water on α-Amino-Acid Crystals and Their Space Groups (SG)^a^

	chiral-resolved crystals l		racemic crystals dl	
α-amino acid	FP (°C)	SG	FP (°C)	SG
a				
valine	–5.6 ± 0.6	*P*2_1_	–9.9 ± 0.8	*P*2_1_/*c*
leucine	–5.5 ± 0.5	*P*2_1_	–8.1 ± 0.5	*P*-1
α-isoleucine	–5.1 ± 0.5	*P*2_1_	–9.8 + 0.6	*P*-1
methionine	–3.7 ± 0.3	*P*2_1_	–7.2 ± 0.1	*P*2_1_/*a*
norleucine	–4.1 ± 0.3	*C*2	–6.7 ± 0.2	*P*2_1_/*a*
*tert*-leucine	–5.8 + 0.6	*P*1	–8.6 ± 0.5	*P*2_1_/*c*
b				
tyrosine	–6.6 ± 0.3	*P*2_1_2_1_2_1_	–1.1 ± 0.2	*Pna*2_1_
alanine	–7.5 + 0.6	*P*2_1_2_1_2_1_	–2.6 ± 0.3	*Pna*2_1_

a (a) α-Amino-acid crystals exhibiting a polar axis for the chiral resolved l-compounds and nonpolar axes for the racemic dl-compounds. (b) The reverse situation holds for the amino-acid crystals listed; the racemic dl-compounds exhibit a polar axis and nonpolar axis for l-compounds. Reproduced with permission from ref (1). Copyright 2021 Wiley.

**Figure 3:**
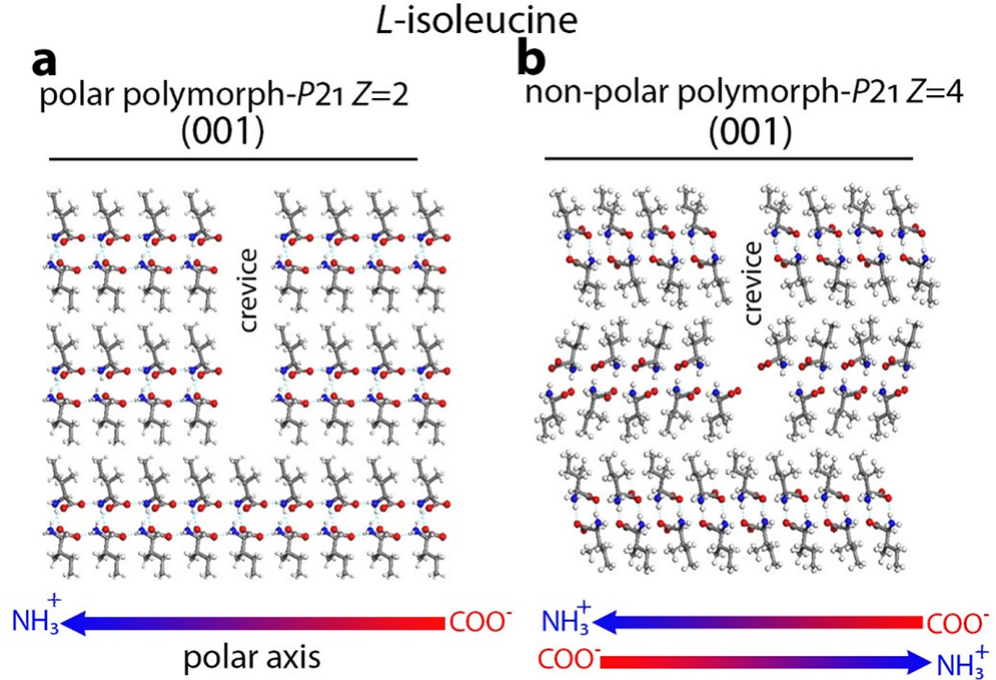
Packing arrangements for the different crevices of l-isoleucine crystals in the (a) polar polymorph space group *P*2_1_ with four molecules in the unit cell, icing temperature −5.1 ± 1.2 °C, and (b) nonpolar polymorph space group *P*2_1_ with eight molecules in the unit cell, icing temperature −8.5 ± 1.2 °C.

Similarly, on the (001) face of the polar polymorph of the isoleucine crystals, Figure 3a, ice crystallizes at a temperature elevated by ∼3 °C in comparison to the very similar, Figure 3b, nonpolar polymorph.^29^

On the basis of these results, two different possible effects were considered to influence the icing of SCW: (I) electrofreezing, given that the polar crystals in a and b of Table 1 belong to the 10 pyroelectric space groups, upon cooling they are charged at their hemihedral faces;^30,31^ (II) geometry, a possible effect resulting from a different alignment of the water molecules within the crevices of the two classes of crystals.

### Freezing Experiments of SCW on Quasi-Amorphous Pyroelectric Surfaces and LiTaO_3_ Crystals

**Figure 4:**
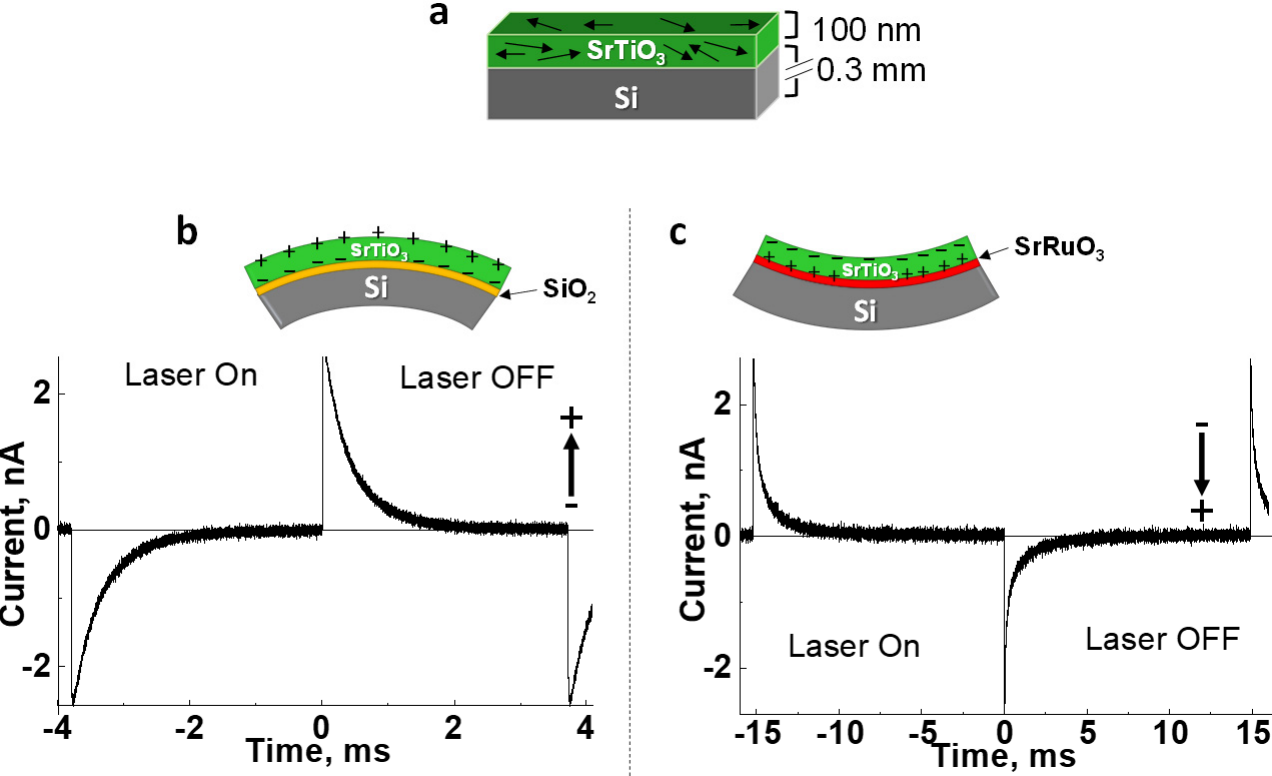
Structures and pyroelectric current of SrTiO_3_ samples. Arrow represents the direction of the polarization in each sample. (a) Nonpyroelectric SrTiO_3_ wafer as a reference. (b) Convex Si\SrTiO_3_ wafer where the pyroelectric response is positive upon cooling. (c) Concave Si\SrRuO_3_\SrTiO_3_ wafer where the pyroelectric response is negative upon cooling. Adapted with permission from ref (32). Copyright 2022 Wiley.

In order to disentangle the electric effect from the geometric effects on heterogeneous ice nucleation of SCW, freezing experiments were performed on positively and negatively charged pyroelectric amorphous, also called quasi-amorphous, thin layers of SrTiO_3_, Figure 4(2,32−34) (see Supporting Information Figures S1 and S2). In these films, the direction of the permanent polarization depends on the direction of the strain gradient expressed as the convex layer is positively charged upon cooling whereas the concave layer is negatively charged. In addition, a nonpyroelectric thin SrTiO_3_ film was used as a reference.

**Figure 5:**
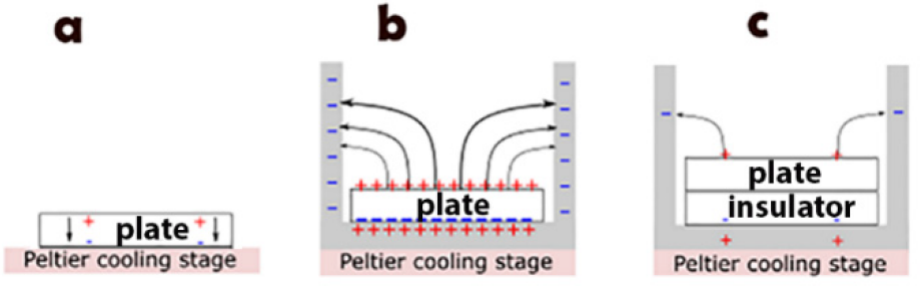
(a) Pyroelectric plate with induced surface charges can be viewed as a parallel plate capacitor where at equilibrium the spontaneous polarization is compensated by external (depolarization) charge. (b) When a pyroelectric plate is placed in a Cu cylinder, the “excess” charge of the bottom side redistributes immediately whereas the charge at the top side requires a much longer equilibration time. (c) When an insulator is placed between the crystal and the Cu cylinder, the electric field, which is developed on the top surface, is effectively suppressed.

**Figure 6:**
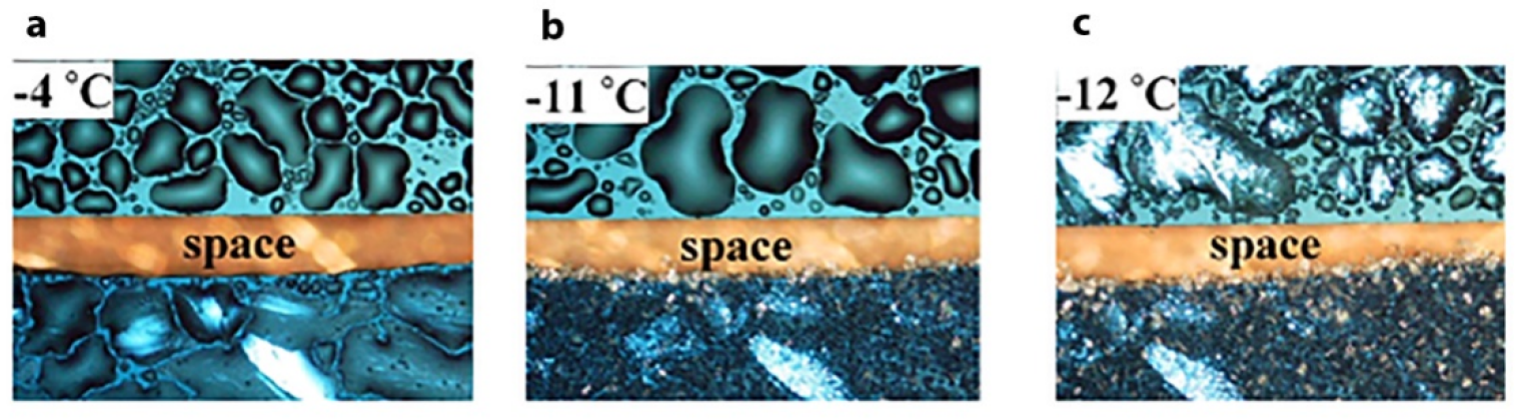
Optical microscopy images of water drops condensed on 7 × 12 mm amorphous (top) and positively charged quasi-amorphous (bottom) films of SrTiO_3_ (100 nm thickness grown on Si) during cooling at (a) −4, (b) −11, and (c) −12 °C. Reproduced with permission from ref (2). Copyright 2021 AAAS.

A pyroelectric plate with induced surface charges can be viewed as a parallel plate capacitor. In such a configuration, the electric field is confined to the plate interior, Figure 5a. In order to perform the icing experiments, those wafers were placed within a copper cylinder on a cooling stage. The copper cylinder has two functions: to ensure the temperature uniformity and to control the electric field produced by the pyroelectric effect. If one of the surfaces is connected to a conductor then its charge will be immediately redistributed and the direction of the electric field will depend on the conductor’s configuration. For the setup depicted in Figure 5b, the top surface of the plate becomes charged with respect to the walls of the copper cylinder with the electric field being close to perpendicular to the surface. Therefore, allowing or prohibiting the charge flow from the bottom surface of the plate to the copper cylinder switches the surface electric field on and off. Accordingly, the freezing of SCW can be compared with and without the field exactly on the same surface. Comparative SCW freezing experiments were performed on either the positively or the negatively charged SrTiO_3_ surfaces and on the nonpyroelectric SrTiO_3_ surfaces in the setup described in Figure 5b. On the positively charged SrTiO_3_ films, ice freezes at −4 ± 1 °C, Figure 6a (bottom), whereas on the nonpyroelectric SrTiO_3_ films, water drops are still liquid at this temperature and freeze at temperatures as low as −12 ± 4 °C, Figure 6a–c (top). On the negatively charged SrTiO_3_ films, the water drops freeze at −19 ± 3 °C.^2^

**Figure 7:**
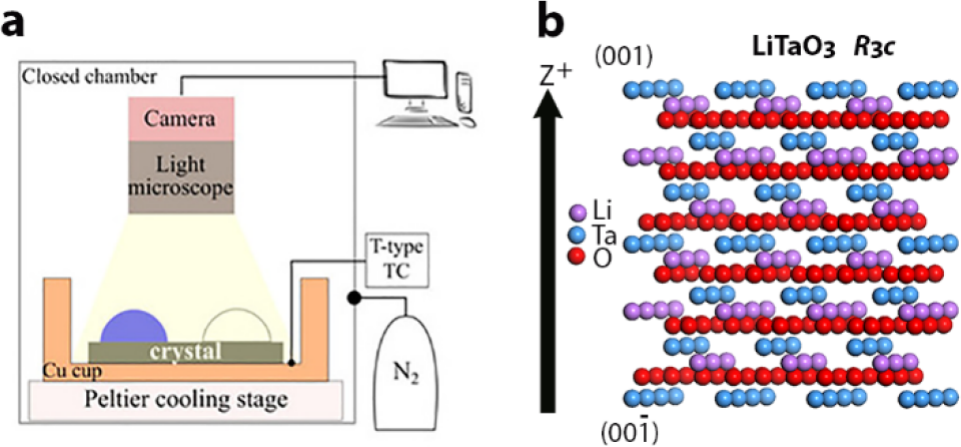
(a) Schematics of the system used for the icing experiments with LiTaO_3_ crystals. (b) Packing arrangement of a pyroelectric LiTaO_3_ crystal displaying the direction of polarity (*z* axis). Two hemihedral faces (001) and (00–1) develop positive and negative charges, respectively, upon cooling.

Similarly, different icing temperatures were observed when the icing experiments were performed on the faces of the pyroelectric LiTaO_3_ crystal_,_Figure 7. On positively charged (001) faces, the drops froze at −7 ± 1 °C and on the negatively charged (00–1) faces at −18 ± 1 °C. Without the field, water freezes at −12.5 ± 3 °C.^4^ X-ray diffraction experiments demonstrated that on the positively charged faces, icing starts at the LiTaO_3_ surface, whereas on the negatively charged surfaces, freezing is initiated at the air/water interface.^2^

Recently, the positive/negative effect on the icing temperature of SCW was also observed on the charged faces of pyroelectric AgI pellets.^25^ Goldberg et al. reported a similar elevation of the icing temperature of SCW at the positively charged surfaces of the following pyroelectric crystals: LiNbO_3_, SrBaNbO_3_, and LiTaO_3._^35^ However, they measured smaller differences in the icing temperatures between the positively and the negatively charged faces in comparison to our results reported on the LiTaO_3_ crystals. After our recent finding that the icing temperature of SCW is triggered by atmospheric CO_2_ (see below), it is suggested that those differences might arise as a result of different environmental conditions present in the two laboratories. In another report, pyroelectric poly(vinylidene fluoride) thin films were used as antifreezing coating materials. In those experiments, it was found that the icing temperature of SCW depends on the polarity of an external field.^36^

### Electrofreezing on Hydrophilic and Hydrophobic Surfaces of Polar Crystals: Role of Ionic Current

**Figure 8:**
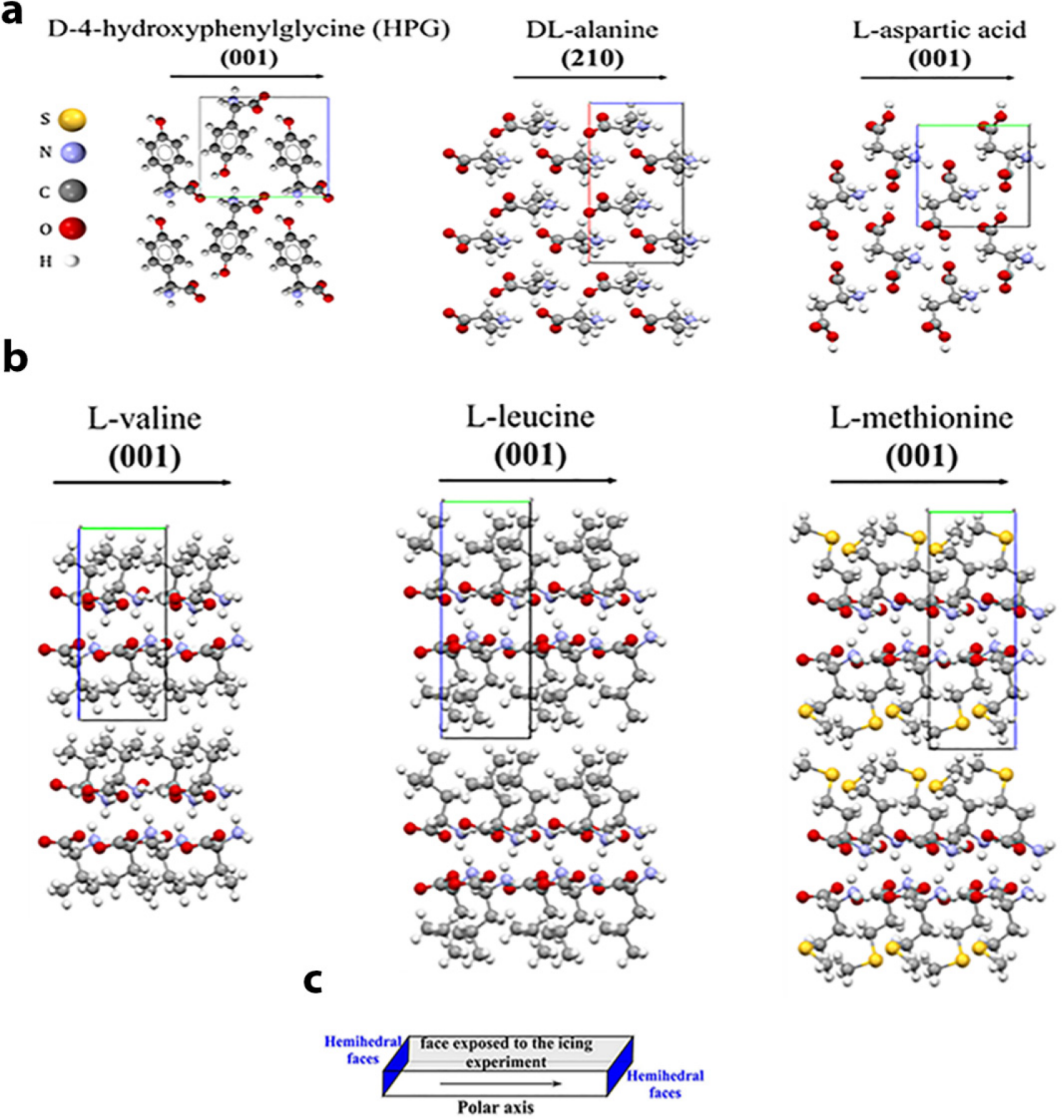
Packing arrangements of polar (a) hydrophilic and (b) hydrophobic α-amino acids. (c) Schematic representation of a plate-like crystal expressing a (001) face on which the icing experiments were performed. Hemihedral faces are shown in blue. Adapted with permission from ref (3). Copyright 2021 Wiley.

**Figure 9:**
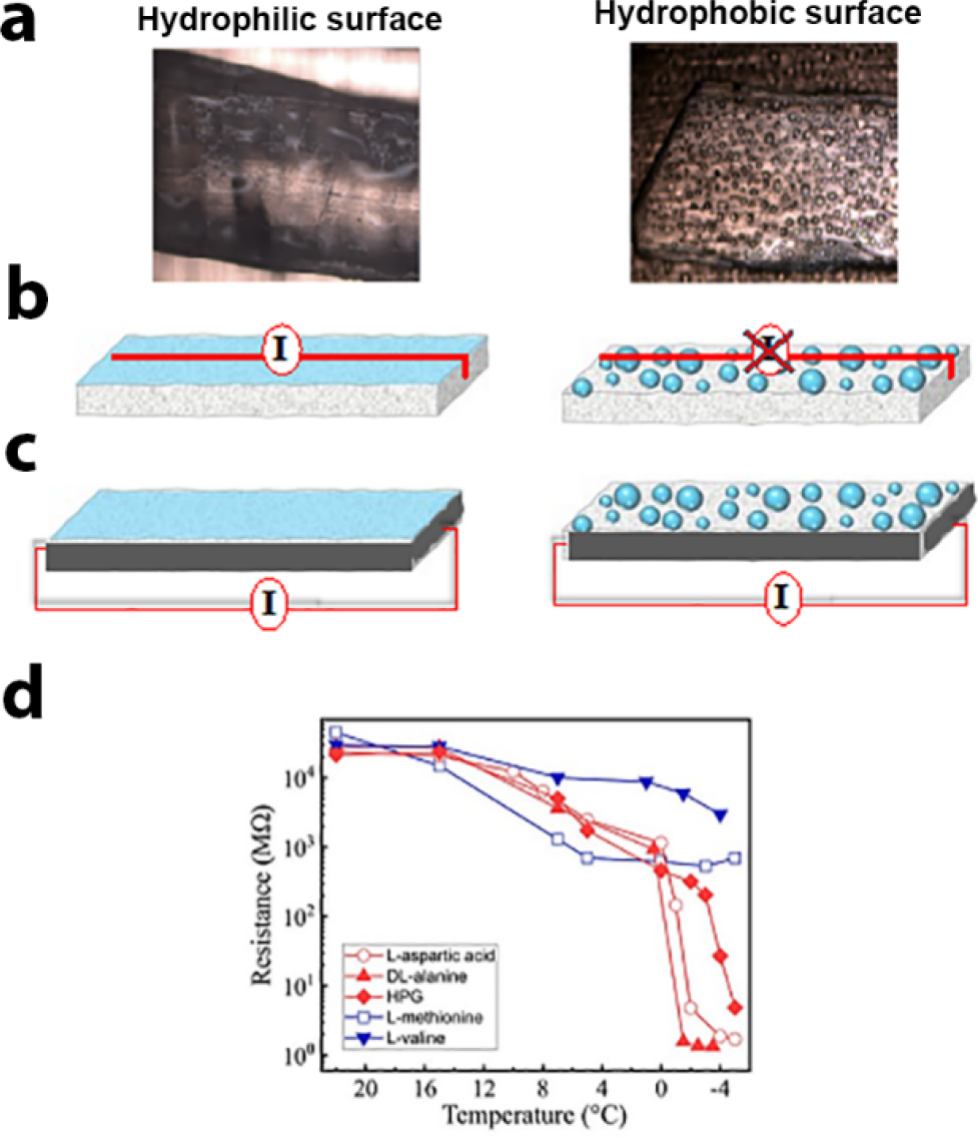
(a) Photos of the hydrated (001) faces of the polar polymorph of (left) hydrophilic l-cysteine and (right) hydrophobic l-methionine. (b) (Left) Continuous water layer on hydrophilic amino acids, inducing ionic current; (right) isolated water drops on the hydrophobic α-amino acids and absence of such current. (c) Redirection of the currents by linking the two hemihedral faces (as in Figure 8c) by a conducting paint (in black). (d) Resistance vs temperature plot as measured (at ac frequency of 1.28 kHz) on the hydrophilic (red) and hydrophobic (blue) α-amino acids. Adapted with permission from ref (3). Copyright 2021 Wiley.

The unexpected positive–negative icing effects on the pyroelectric surfaces, described above, led to the conjecture that electrofreezing of SCW might be influenced by compensating charges attracted from the environment during the cooling process. It was expected that those charges might be attracted from water residing at the different faces that delineated the crystals. To support this hypothesis, icing experiments were performed on well-expressed hydrophilic and hydrophobic surfaces of α-amino acids, residing parallel to the polar axis. The crystal structures of some of the hydrophilic and hydrophobic crystals investigated are shown in Figure 8. The icing temperatures on the faces of the hydrophilic crystals placed on an insulator were higher by 3–5 °C in comparison to the case with a conductor (placed on a Cu plate or painted on the bottom with a conducting paint). This difference was explained by showing that the hydrophilic faces are wetted during cooling and created a continuous water layer between the hemihedral faces (Figure 9a–c).

Resistance measurements demonstrated the occurrence of an ionic current created by compensating charges in the water layer, which are attracted by the charged hemihedral faces, Figure 9d (red). By linking the two hemihedral faces with a conductor, this current was redirected away from the water layer and the difference in the freezing temperature was eliminated.

Further support for this deduction was obtained by the performance of comparative icing experiments on top of analogous faces of hydrophobic α-amino acids. In contrast to the hydrophilic crystals, on the hydrophobic surfaces, the wetting resulted in isolated droplets. As a result, an ionic current is not created on those surfaces, as confirmed by resistance measurements, Figure 9d (blue). The icing temperature on those faces was the same when the hydrophobic crystals were placed on a conductor or on an insulator Figure 9.

The discovery of such current refutes our previous speculation that the role of the conductor was to annul the pyroelectric field within the crevices of the polar crystals.^21^

**Figure 10:**
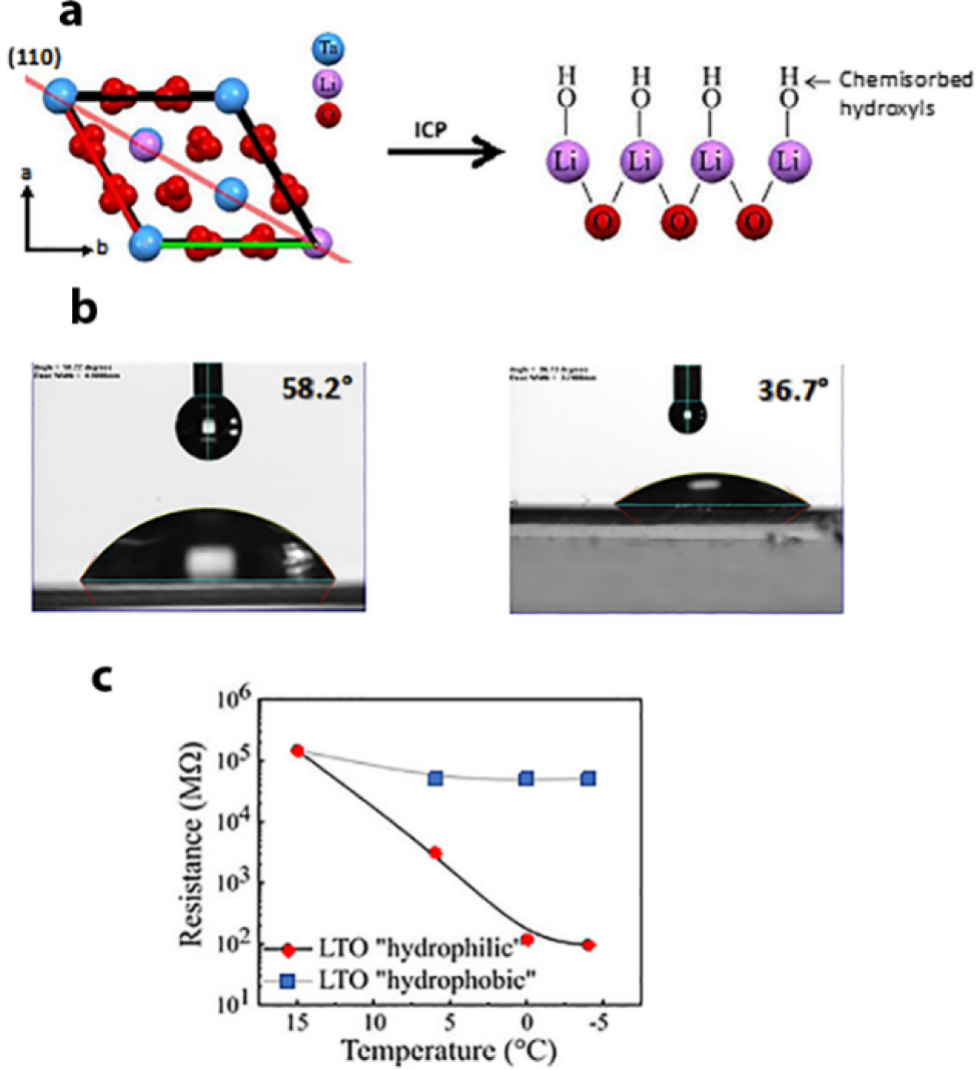
(a) Packing arrangement of LiTaO_3_ showing the (110) face as a red line and the chemistry change resulting from plasma treatment. (b) Contact angles of water drops before and after plasma treatment. (c) Resistance vs temperature plots measured. Adapted with permission from ref (3). Copyright 2021 Wiley.

A similar hydrophilic–hydrophobic effect was found by performing the icing experiments on the insoluble pyroelectric LiTaO_3_ crystal. Its (110) face, Figure 10a, which resides parallel to the polar axis, has a contact angle of 58°, Figure 10b. However, by application of an inductive plasma treatment it is converted to be more hydrophilic (contact angle 37°),^37^Figure 10b. The resistance below the dew point on the more hydrophobic surfaces was higher (∼10^5^ MΩ) in comparison to the more hydrophilic surfaces (∼10^2^ MΩ), Figure 10c.

Parallel icing experiments performed on the more hydrophilic face resulted in a freezing temperature of −9.4 vs −14.2 °C on the untreated face. This difference is induced by the ionic current and not by the coarsening of the surface as a result of the application of the plasma since the difference in the freezing temperature was eliminated by linking the two hemihedral faces with a conducting paint.^3,38^

### Role Played by Atmospheric CO_2_ in Electrofreezing of SCW

**Figure 11:**
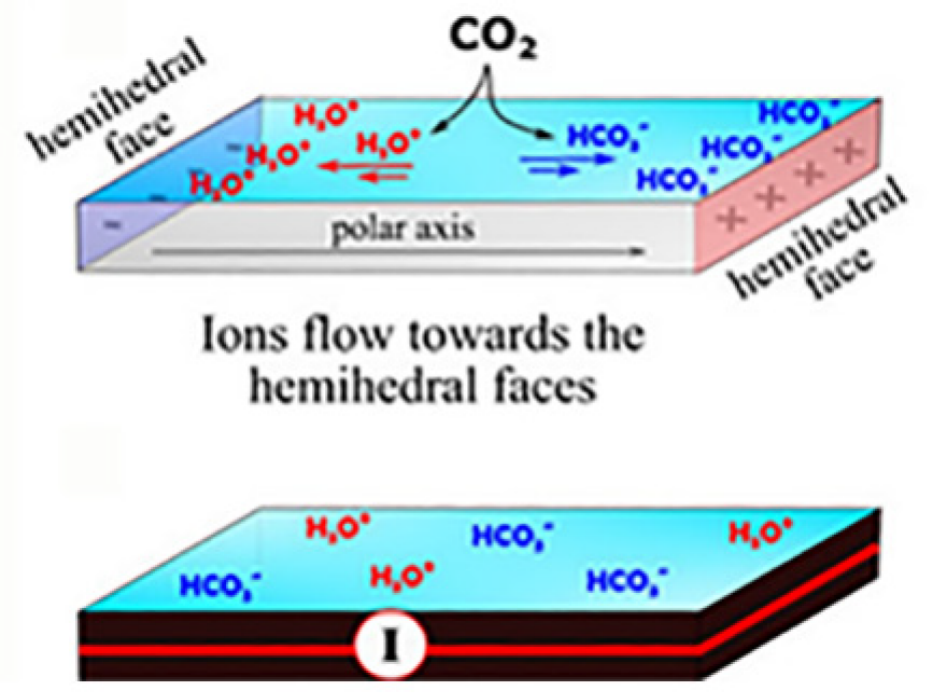
Creation of bicarbonate ions and their attraction by the hemihedral faces. Reproduced with permission from ref (3). Copyright 2021 Wiley.

The results of the above experiments implied that the compensating ions that created the electric current on the hydrophilic surfaces should be responsible for the electrofreezing effect. The most common ion present in water is bicarbonate, created by the inevitable dissolution of atmospheric CO_2_ in water, Figure 11. This surmise was confirmed experimentally by performing comparative icing experiments on the hydrophilic faces of pyroelectric α-amino-acid crystals using pure water pH 7 and pure water saturated with CO_2_ by dissolution of dry ice, pH 4.^3^ The icing temperatures, as shown in Table 2, were higher with dissolved CO_2_ (pH 4) in comparison to pure water. In the absence of the ionic current, by applying the conductive paint, the icing temperatures of the water drops were the same in the presence and absence of CO_2_. In addition, the experiments on the hydrophobic surfaces resulted in no differences in the freezing temperature of pH 7 and 4 water droplets.^3^

**Table 2:** Icing Temperatures (°C) Measured on Faces Parallel to the Polar Axis of the Hydrophilic α-Amino-Acid Crystals in the Presence and Absence of a Pyroelectric Field, i.e., without (*T*_clean_) and with Conductive Paint (*T*_painted_) of Condensed Water Drops of pH 7 and 4^a^

	*T*_clean_	*T*_painted_	Δ*T*
pH 4			
HPG	–5.8 ± 0.8	–11.0 ± 1.7	5.2 ± 0.9
dl-alanine	–1.5 ± 0.7	–4.7 ± 0.8	3.2 ± 0.5
l-aspartic acid	–4.8 ± 1.2	–8.3 ± 0.4	3.5 ± 0.5
pH 7			
HPG	–11.3 ± 2.8	–11.4 ± 1.7	0.1 ± 1.6
dl-alanine	–5.1 ± 0.2	–5.3 ± 0.2	0.2 ± 0.1
l-aspartic acid	–8.6 ± 0.6	–8.3 ± 0.9	–0.3 ± 0.5

a HPG denotes d-4-hydroxy-phenylglycine.

l

Similar results are obtained with the hydrophilic crystals of the polar polymorph of cysteine,^3^ the mixed crystal of l-asparagine monohydrate/l-aspartic acid,^21^ and a polar crystaline crust of dl-tyrosine.^3^ should be monohydrate/l-aspartic

In order to differentiate between the role played by the bicarbonate and that of the hydronium ions, the icing experiments were performed directly on the two positively and negatively charged (001) and (00–1) hemihedral faces of the LiTaO_3_ crystals.

**Figure 12:**
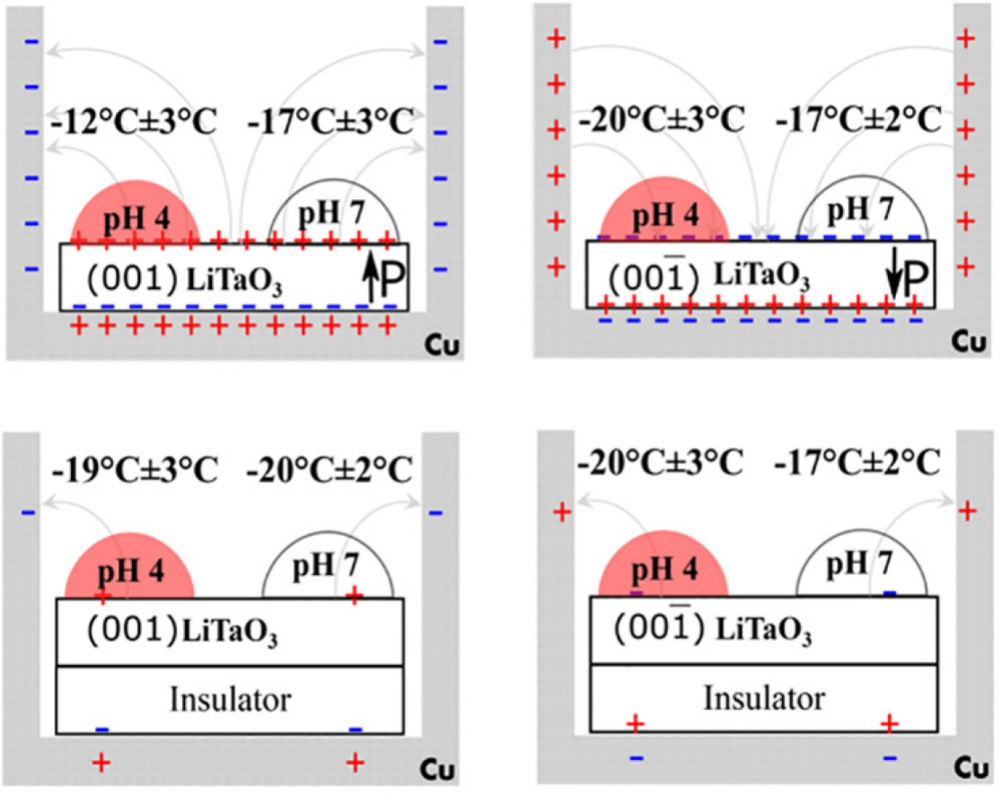
Summary of comparative icing experiments with drops of 30 μL, containing bicarbonic acid, deposited on the positive (001) and negative (00–1) faces of LiTaO_3_ in the presence and absence of the insulators.

On the positively charged (001) face, upon cooling, the droplets containing bicarbonate ions freeze at −12 ± 2 °C, higher by 5 °C in comparison to the pure water drops (−17 ± 2 °C), Figure 12. On the negatively charged (00–1) face, the droplets containing a high concentration of hydronium ions freeze at the same or slightly lower temperature in comparison to pure water.

The augmentation of the icing temperature within dilute aqueous solutions depends on the structure and composition of the ions. Consequently, di- or polyvalent acids and salts, which can result in various associated ionic structures, might not impact electrofreezing.^39,40^

It should be pointed out that the ions have a path to reach the charged surface even on hydrophobic surfaces when the experiments are performed directly on the hemihedral faces, in contrast to those performed on the faces parallel to the polar axes. In such cases, the ions are attracted to the hemihedral face, and therefore, such surfaces can be either hydrophilic or hydrophobic as shown in the case of the icing experiments directly performed on the hydrophobic hemihedral faces of LiTaO_3_.

**Figure 13:**
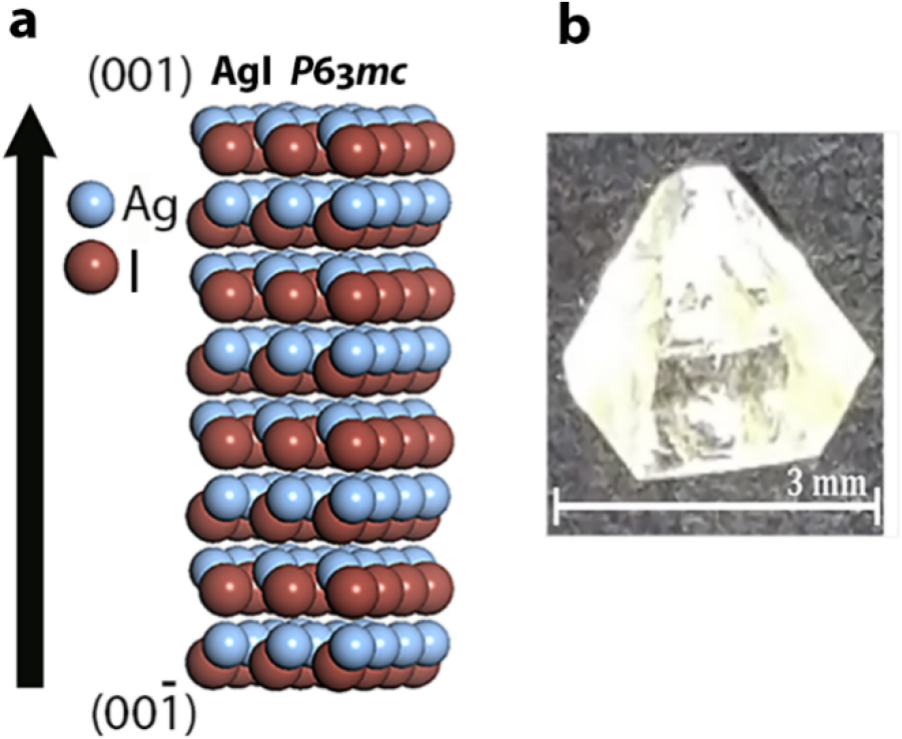
(a) Crystal structure of the polar polymorph of AgI. Arrow indicates the direction of the polar axis and hemihedral faces labeled (001) and (00–1). Crystals were cleaved prior to the icing experiments. (b) Single crystal of AgI.

In the case of the (001) face of AgI crystals, Figure 13, where the Ag^+^ ions reside, the icing temperature of pH 4 drops was higher by 1.6 ± 0.4 °C in comparison to the pure water drops (pH 7). In the presence of the insulator, the temperature decreases to −3.5 ± 0.3 °C. This was also the icing temperature on the negative face of the crystal, where the I^–^ ions reside, both in the presence and in the absence of the pyroelectric field, Table 3.^41−44^

**Table 3:** Icing Experiments on the (001) and (00–1) Faces of Cleaved Crystals of AgI^a^

cleaved wurtzite AgI single crystal		pH 4	pH 7
with pyroelectric charge	Ag^+^ side	–1.9 ± 0.4	–3.5 ± 0.3
	I^–^ side	–3.3 ± 0.3	–3.4 ± 0.2
without pyroelectric charge	Ag^+^ side	–3.4 ± 0.2	–3.5 ± 0.5
	I^–^ side	–3.5 ± 0.3	–3.7 ± 0.2

a Three cases were examined (i) with CO_2_ pH 4, (ii) with pure water pH 7, and (iii) in the presence and absence of the pyroelectric field.

Although the measured differences were small, they could be reliably determined since the energy barrier for icing formation near the melting point is extremely large per degree.^45^ Therefore, each degree of supercooling is significant. Since the cooling rate in all experiments was 2 °C/min., there was a distinct time interval of ∼45 s between the icing of the two drops.^3^

### Electrofreezing of SCW As Influenced by Ice-Maker and Ice-Breaker Ions

**Figure 14:**
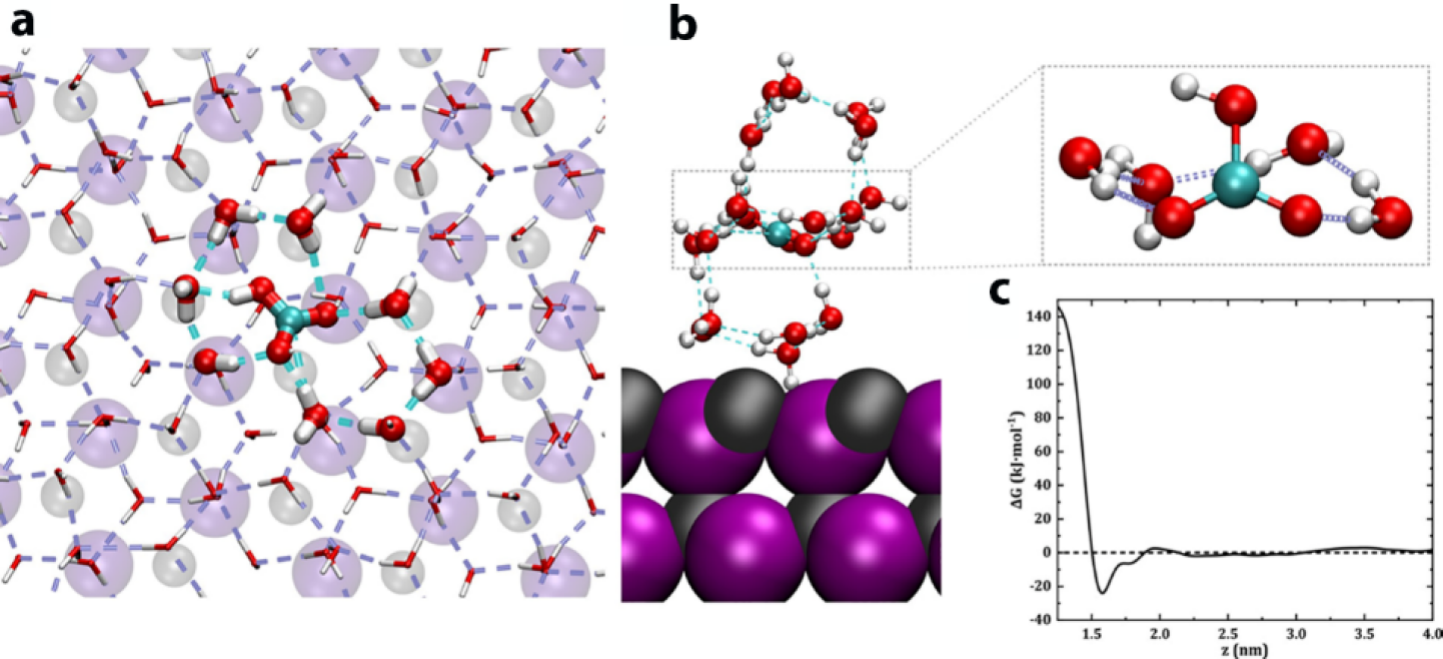
MD results for HCO_3_^–^ incorporation into the ice: (a) view down the (001) ice face showing the inclusion of HCO_3_^–^ into the ice structure; (b) close up of the included ion within the second layer of ice formed on the AgI surface; (c) free energy profile of the HCO_3_^–^ ion at the AgI interface in the liquid phase in the *z* direction of the surface normal. Reproduced with permission from ref (4). Copyright 2021 Wiley.

Molecular dynamics simulations of bicarbonate ions on the surfaces of the polar polymorph of AgI were performed by Harries and Allolio.^4^ It was found that once the bicarbonate ion is inserted within the second water layer, near the Ag^+^ ions, icing immediately commences. No such effect occurs beyond the third layer. It was also found that near the crystal surface, a HCO_3_^–^ ion is included within the hexagonal ice ring, generated by epitaxy, through the replacement of hydrogen bonds by covalent bonds. Such replacement does not result in a significant strain, since the O···O···O angle of the water hexagons which incorporate the HCO_3_^–^ differs only by 2° from the 109.4° present in the pure water molecule hexagons, Figure 14. Such replacement stabilizes the embryonic ice nuclei and consequently raises the icing temperature of SCW. The MD simulations were also performed near the (00–1) surface of the AgI, where the I^–^ ions reside and the water did not freeze regardless of the presence of the ions.

**Figure 15:**
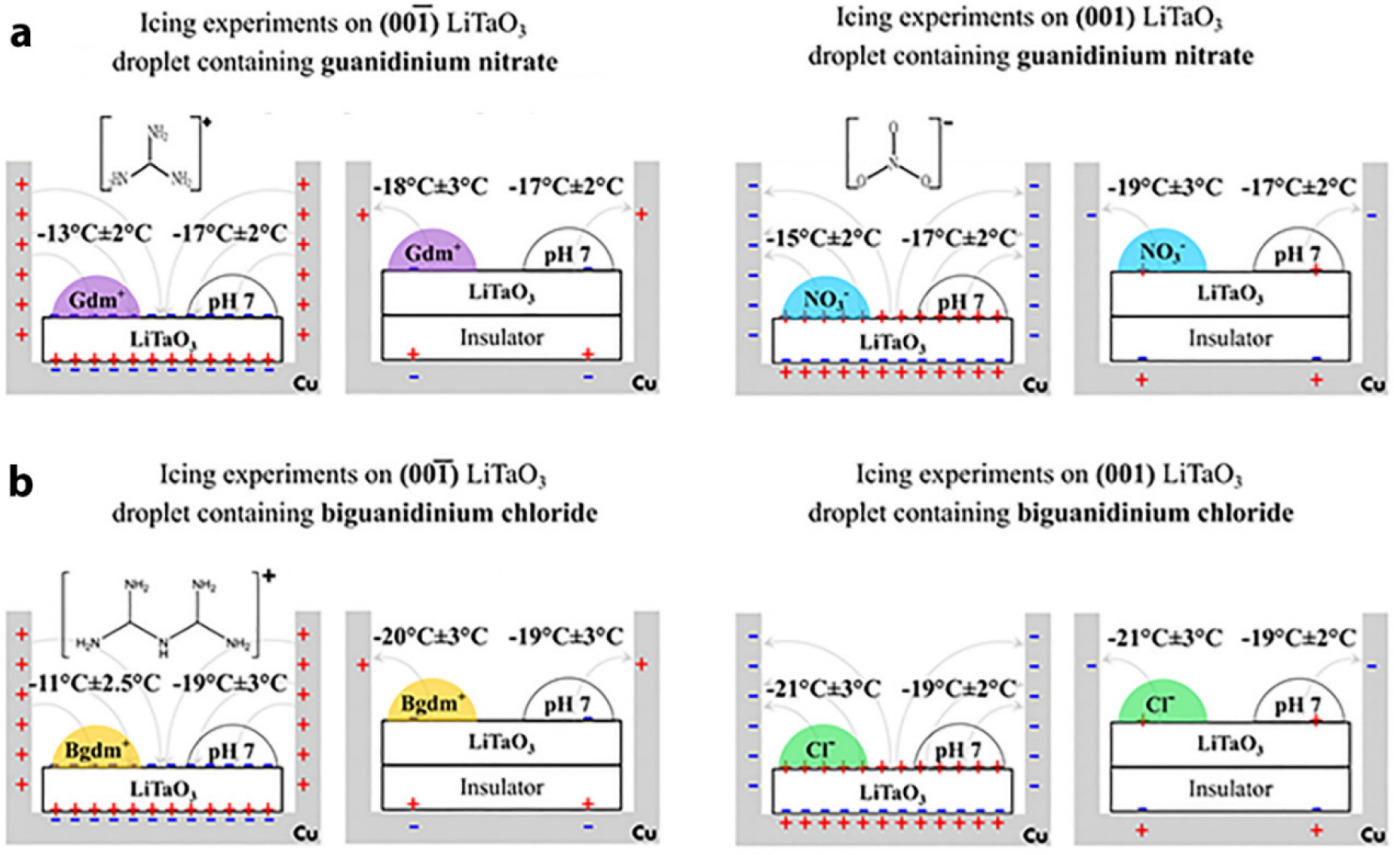
Summary of comparative icing experiments with 30 μL drops of (a) guanidinium nitrate and (b) biguanidinium chloride deposited on the positive (001) and negative (00–1) faces of LiTaO_3_ in the presence and absence of the insulators. Reproduced with permission from refs (4) and (40). Copyright 2021 and 2022 Wiley and American Chemical Society.

**Figure 16:**
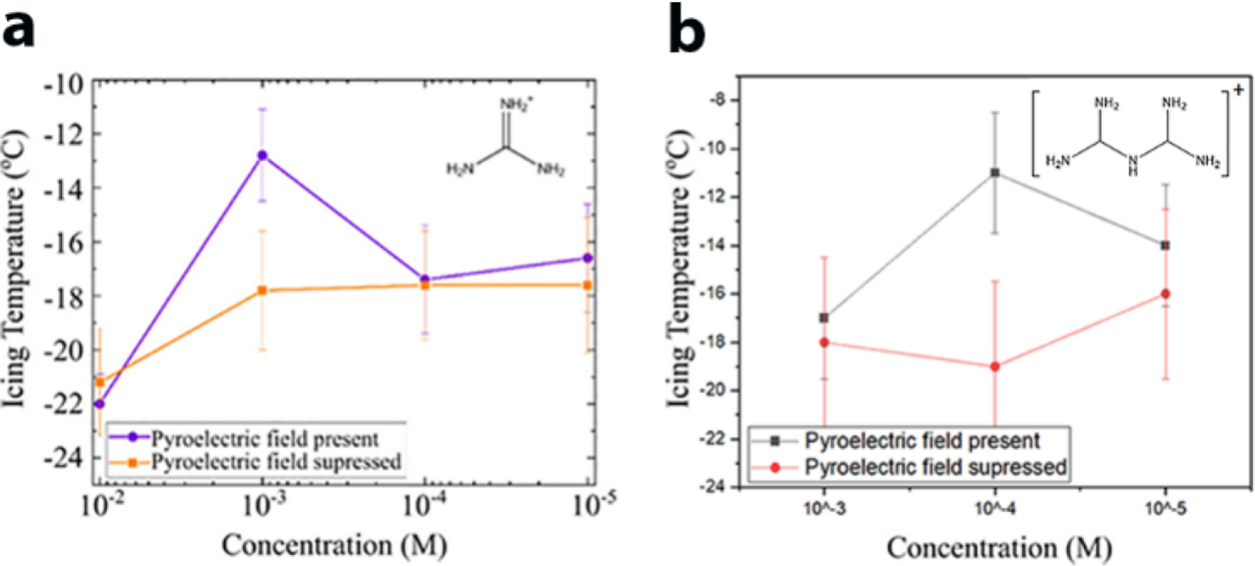
Icing temperature as a function of the concentration of (a) guanidinium^+^ and (b) biguanidinium^+^ ions present in solution. Reproduced with permission from refs (4) and (40). Copyright 2021 and 2022 Wiley and American Chemical Society.

From the MD calculations it follows that the elevated freezing temperature of SCW is due to the planar trigonal configuration of the bicarbonate ions which assemble with water molecules to form “ice-like” assemblies. This simulation suggested that similar trigonal planar ions and other ions that have the tendency to interact with water molecules to form such assemblies should augment also the icing temperature of SCW. Indeed, two analogous nitrogenous trigonal planar analogues of carbonic acid, i.e., NO_3_^–^ and guanidinium^+^ (Gdm^+^),^4^ and biguanidinium^+^ (Bgdm^+^),^40^ structures shown in Figure 15 and 16, which possess this tendency should augment the icing temperature. Ions of different configurations, such as Cl^–^ and SO_4_^2–^, in contrast, should reduce the icing temperature. This was confirmed experimentally, as summarized in Figure 15.

Ions dissolved in solution generally reduce the icing temperature of SCW; therefore, the elevation of the icing temperature by electrofreezing should be limited to specific concentrations. The concentrations of the bicarbonates obtained by the dissolution of atmospheric CO_2_ is in the regime required for the elevation of the icing temperature. Experiments with other trigonal ions and with biguanidinium^+^ ions were performed with different ion concentrations. The optimal concentration for ions of Gdm^+^ and NO_3_^–^ was found to be 10^–3^ M and for Bgdm^+^ 10^–4^ M, Figure 16. At these concentrations, Gdm^+^ and Bgdm^+^, attracted to the negative face, elevate the icing temperatures by ∼5 and ∼8 °C, respectively.^4,40^

Quantitative understanding of the required effective concentrations for each ice-maker ion and the temperatures at which they augment the icing temperatures should require detailed theoretical simulations.

**Figure 17:**
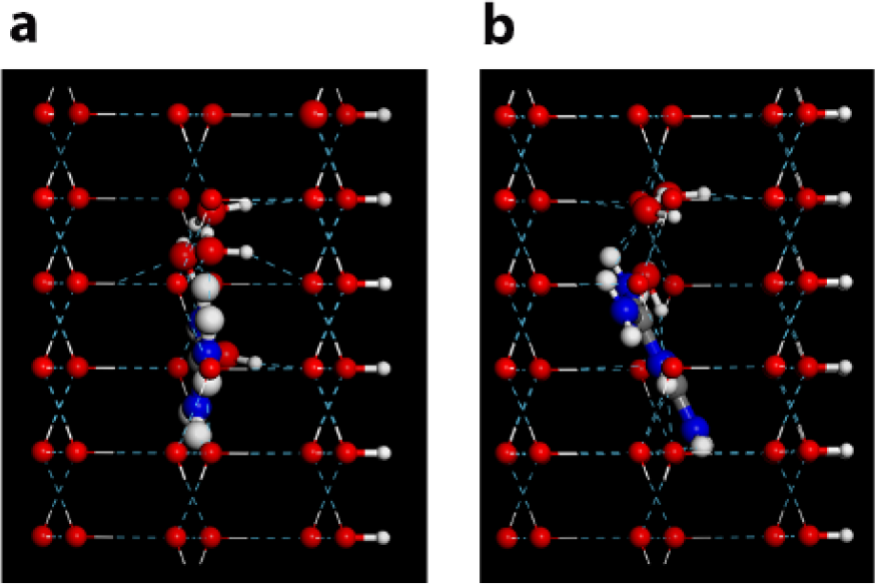
Oriented proton-ordered hexagonal ice cluster embedding one molecule of the additive: (a) Gdm^+^ ion and (b) Bgdm^+^ ion. Each molecule is shown from the side view with the polar axis direction pointing to the right. Color code: O, red; H, white; N, blue; C, gray. Reproduced with permission from ref (40). Copyright 2022 American Chemical Society.

Comparison between the incorporation of Gdm^+^ vs Bgdm^+^ ions in the hexagonal ice structure using the potential energy computations calculated with the Material Studio package indicated that ice clusters incorporating Bgdm^+^ yield a lower energy by 27 kcal/mol in comparison to those incorporating Gdm^+^, in agreement with the experimental results, Figure 17.

**Figure 18:**
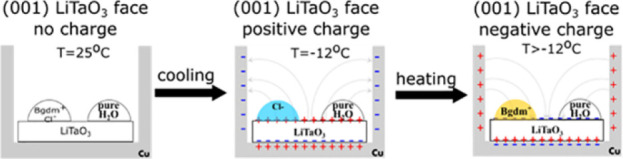
Icing of SCW by heating aqueous solutions containing Bgdm^+^Cl^–^. Reproduced with permission from ref (40). Copyright 2022 American Chemical Society.

One of the distinct properties of pyroelectric crystals is that the electric charges created on the same hemihedral face can be interchanged depending on whether the crystal is cooled or heated. This unique property has been used in order to disentangle completely the electric effect from any geometric effect as illustrated here with the Bgdm^+^Cl^–^ aqueous solutions.^40^ Water drops were deposited on the (001) face of the LiTaO_3_ crystals, which are positively charged. The crystals were cooled to −12 °C without freezing, since Bgdm^+^ ions are not attracted to the positively charged surface. The cooling was halted, and the positive charge was allowed to dissipate. Consequently, heating the specimen (2 °C/min) from −12 to −8 °C created a negative charge at that surface and attracted the Bgdm^+^ ions, which accelerate freezing, Figure 18.^40^ In contrast, if those crystals are cooled then the water drops freeze below −17 °C. Similar icing experiments by heating were described on the (00–1) face of LiTaO_3_ crystals.^2^

### Role of Proton-Ordered Water Molecules within the Crevices of Polar α Amino-Acid Crystals

The higher icing temperature measured within the crevices of the polar hydrophobic crystals vs the nonpolar analogues, Table 1, and on the two polymorphs of isoleucine crystals, Figure 3, implied that there must be an additional effect that influences the augmentation of the icing temperature on surfaces of polar crystals. The water molecules within a crevice of polar crystals delineated by the positively and negatively charged NH_3_^+^ and CO_2_^–^ walls should be aligned in a proton-ordered mode. In contrast, within the analogous nonpolar crystals, where each wall of the crystal is composed of alternative NH_3_^+^ and CO_2_^–^ groups, the water molecules should be less oriented. Such proton ordering has been considered to decrease the free energy of heterogeneous ice nucleation and thus can be responsible for the additional reduction of the icing temperature.^1,46^

**Figure 19:**
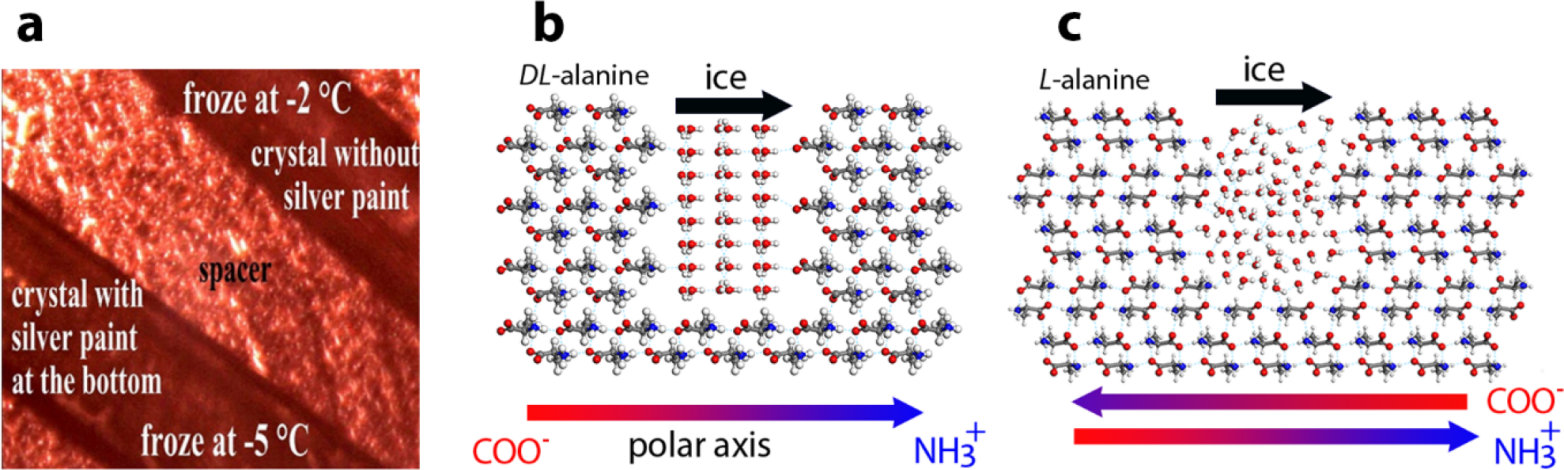
(a) Icing temperatures measured on dl-alanine crystals in the presence and absence of the conductive paint. Reproduced with permission from ref (21). Copyright 2022 American Chemical Society. (b and c) Model of the alignment of proton-ordered hexagonal ice crystals within crevices of the polar dl-alanine and nonpolar l-alanine crystals.^21^

Accordingly, the augmentation of the icing temperature on top of the hydrophilic polar surfaces was demonstrated to result from both the pyroelectric effect and the proton ordering of the water molecules within its crevices. The operation of those two effects is demonstrated by the comparative icing experiments of the crystals of dl- and l-alanine, Figure 19.

The pyroelectric influence on the icing temperature of dl-alanine was removed by performing the icing experiments on its hydrophilic face in the presence of the conducting paint, Figure 19a. The presence of the conductive paint reduces the icing temperature from ∼−2.5 to ∼−5 °C, and this difference is due to the bicarbonate ions. Within the crevices of the polar crystals, the icing temperature is higher than the icing temperature found on the surfaces of the analogous nonpolar l-alanine crystals, Figure 19c, which is ∼−7.5 °C, Table 1.^1^ The interaction energies per water molecules of a single ice-like (0001) bilayer within the crevices of were calculated to be −3.8 kcal/mol for dl-alanine and −2.9 kcal/mol for l-alanine.^1^ This deduction is also in keeping with recent MD simulations by Zhou et al.^46^ that suggested that hydrogen polarity regulates heterogeneous ice nucleation.

In conclusion, icing of SCW can be influenced by epitaxy^26,47−49^ or other factors.^6,50,51^ Electrofreezing of SCW on polar surfaces of pyroelectric crystals uncovered two additional independent effects. (a) The first effect is a chemically driven process induced by the pyroelectric effect which encompasses the attraction of compensating ions from the water environment. Two classes of ions were found: those that interact with water to create ice-like assemblies that trigger icing and those that interfere with the formation of such nuclei. (b) The second effect is the alignment of the water ordering within crevices of polar crystals, which reduces the free energy of ice nucleation. On polar surfaces, such as AgI crystals, the elevated icing temperature is affected by epitaxy,^26^ by compensating ions,^4^ and by the proton-ordering effect.^1,46^

We anticipate that the mechanism of the electrofreezing of SCW within an electrolytic cell should also include a chemical component. The role of different electrodes on electrofreezing is currently under investigation.

## Supporting Information Available

Preparation and properties of quasi-amorphous materials and schematic representation of the strain developing in the Si/SrTiO_3_ and Si/SrRuO_3_/SrTiO_3_ structures (PDF)
