# Supplementary material for: Chemical Nature of Heterogeneous Electrofreezing of Supercooled Water Revealed on Polar (Pyroelectric) Surfaces
Source: Acc Chem Res. 2022 May 3;55(10):1383–94. doi: 10.1021/acs.accounts.2c00004 (PMC9118552; doi:10.1021/acs.accounts.2c00004)
Supplement: Supplementary file 1 — ar2c00004_si_001.pdf [file ar2c00004_si_001.pdf]

Supporting Information

The Chemical Nature of Heterogeneous Electrofreezing of Supercooled Water Revealed on Polar (Pyroelectric) Surfaces

Leah Fuhrman Javitt, Sofia Curland, Isabelle Weissbuch, David Ehre,*

Meir Lahav and Igor Lubomirsky**

david.ehre@weizmann.ac.il meir.lahav@weizmann.ac.il igor.lubomirsky@weizmann.ac.il

Department of Molecular Chemistry and Materials Science, Weizmann Institute of Science, Rehovot, 7610001, Israel

Quasi-amorphous materials:

Quasi-amorphous materials are non-crystalline polar materials (Ref. 3), which exhibit a large pyroelectric effect. In these materials, the transformation from the non-polar form to the polar form does not require crystallization, which means that the surface structure of the non-polar amorphous film and the polar quasi-amorphous film are similar. This surface similarity allows us, by comparing the freezing of water on non-polar amorphous film and the polar quasi-amorphous film, to investigate the way polarity and pyroelectricity affect ice nucleation. The preparation of quasi-amorphous $SrTiO_3$ films is composed of two steps. First, a thin amorphous film (50–200nm) of $SrTiO_3$ is deposited by RF sputtering on a substrate (Si, SiO_2, or Si covered with a $SrRuO_3$ layer), (refs. 1-4). The film is then pulled at a rate of 1–5mm/h through a narrow (1–3mm) hot zone with a peak temperature of 550–650 °C (Figure S1) (Ref. 4) to transform the non-polar amorphous film to a polar quasi-amorphous film. Characterization techniques like transmission electron microscopy (TEM), X-ray diffraction (XRD) were used to verify the absence of crystallization.

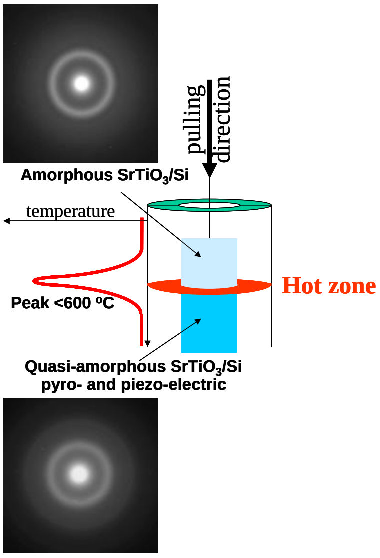

Figure S1. Preparation of a quasi-amorphous film and electron diffraction patterns of the amorphous and quasi-amorphous films. Reproduced with permission from Ref. 4. Copyright 2022 Wiley.

The temperature gradient plays an essential role in the transformation of the amorphous into the quasi-amorphous phase. The resulting in-plane strain gradient produces the following effects: (i) suppression of crystallization, even in the presence of voids in the as-deposited films; and (ii) formation of a permanent electric polarization due to flexoelectric coupling. All as-deposited voids must be eliminated in order to suppress crystallization. These voids are closed by shear stress, which is developing as the sample moves through the temperature gradient due to the expansion of the film as a result of the change from the high density of the as-deposited amorphous phase to the low-density quasi-amorphous phase. The polarity of the quasi-amorphous phase is also related to the strain gradients that develop in the amorphous film during heat treatment. (Ref. 5) Not only the magnitude but also the direction of the permanent electric polarization, as monitored by pyroelectric measurements, depends on the sample structure as shown in Figure 4 of the article. (Ref 1) These strain gradients are able to align microscopic electric dipoles. The poling and resulting polarization are retained even after the strain gradient is removed. The direction of the polarization is determined by the direction of strain gradient as illustrated in Figure S2. (Ref. 1)

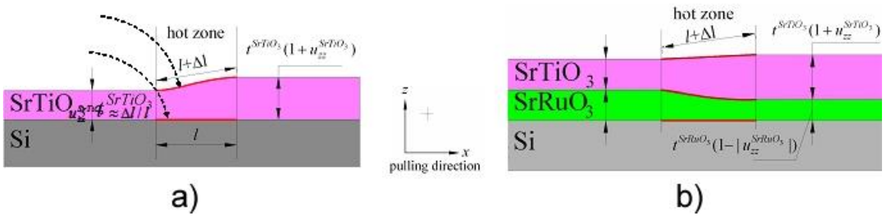

Figure S2. Schematic representation of the strain developing in the Si\$SrTiO_3$ and Si\$SrRuO_3$\$SrTiO_3$ structures during pulling through the temperature gradient. The red lines indicate the interfaces at which the in-plane strain is considered. Reproduced with permission. from Ref.1 Copyright 2022 Wiley.

References:

1. Shelukhin, V.; Ehre, D.; Lavert, E.; Wachtel, E.; Feldman, Y.; Tagantsev, A.; Lubomirsky, I.: Structural Determinants of the Sign of the Pyroelectric Effect in Quasi-Amorphous SrTiO3 Films. *Adv. Funct. Mater.* **2011**, *21*, 1403-1410.

2. Ehre, D.; Cohen, H.; Lyahovitskaya, V.; Lubomirsky, I.: X-ray photoelectron spectroscopy of amorphous and quasiamorphous phases of Ba Ti O 3 and Sr Ti O 3. *Phys. Rev. B* **2008**, *77*, 184106.

3. Ehre, D.; Lyahovitskaya, V.; Tagantsev, A.; Lubomirsky, I.: Amorphous Piezo-and Pyroelectric Phases of BaZrO3 and SrTiO3. *Adv. Mater.* **2007**, *19*, 1515-1517.

4. E. Wachtel and I. Lubomirsky, Quasi-Amorphous Inorganic Thin Films: Non-Crystalline Polar Phases, *Adv. Mater.* **2010**, 22, 2485–2493

5. Marcolli, C.; Nagare, B.; Welti, A.; Lohmann, U.: Ice nucleation efficiency of AgI: review and new insights. *Atmos. Chem. Phys.* **2016**, *16*, 8915-8937.
